# Humpback whales (*Megaptera novaeangliae*) attend both Mexico and Hawaii breeding grounds in the same winter: mixing in the northeast Pacific

**DOI:** 10.1098/rsbl.2021.0547

**Published:** 2022-02-16

**Authors:** James D. Darling, Katherina Audley, Ted Cheeseman, Beth Goodwin, Edward G. Lyman, R. Jorge Urbán

**Affiliations:** ^1^ Whale Trust, Makawao, HI 96768, USA; ^2^ Whales of Guerrero, Barra de Potosi, Guerrero 40830, Mexico; ^3^ Happywhale, Santa Cruz, CA 95060, USA; ^4^ Marine Ecology Research Centre, Southern Cross University, Lismore, Australia; ^5^ Eye of the Whale Marine Mammal Research, Kamuela, HI 96743, USA; ^6^ Hawaiian Islands Humpback Whale National Marine Sanctuary, Kihei, HI 96753, USA; ^7^ Departamento de Ciencias Marinas y Costeras, Universidad Autónoma de Baja California Sur, La Paz, B.C.S. 23080, México

**Keywords:** humpback whales, Hawaii, Mexico, population definition, mixing, breeding grounds

## Abstract

Humpback whales that assemble on winter breeding grounds in Mexico and Hawaii have been presumed to be, at least, seasonally isolated. Recently, these assemblies were declared Distinct Population Segments under the US Endangered Species Act. We report two humpback whales attending both breeding grounds in the same season—one moving from Hawaii to Mexico and the other from Mexico to Hawaii. The first was photo-identified in Maui, Hawaii on 23 February 2006 and again, after 53 days and 4545 km, on 17 April 2006 in the Revillagigedo Archipelago, Mexico. The second was photo-identified off Guerrero, Mexico on 16 February 2018 and again, 49 days and 5944 km later, on 6 April 2018 off Maui. The 2006 whale was identified in summer off Kodiak Island, Alaska; the 2018 whale off British Columbia. These Mexico–Hawaii identifications provide definitive evidence that whales in these two winter assemblies may mix during one winter season. This, combined with other lines of evidence on Mexico–Hawaii mixing, including interchange of individuals year to year, long-term similarity of everchanging songs, one earlier same-season travel record, and detection of humpback whales mid-ocean between these locations in winter, suggests reassessment of the ‘distinctiveness' of these populations may be warranted.

## 1. Introduction

Humpback whales in the North Pacific migrate between high latitude summer feeding grounds around the Pacific Rim and winter calving/breeding grounds in tropical waters [1,2]. Two well-known winter grounds are (i) in the eastern North Pacific, the waters off Mexico, both near shore, along the Baja California Peninsula and the mainland coasts, and offshore around the Revillagigedo Archipelago [3], and (ii) in the central North Pacific, in the waters around the Hawaiian Islands [4]. These regions are separated by 4500–6000 km. Whales may be present in these regions from November through May, with peak numbers in February and March.

Largely owing to distance of separation, it has been presumed that the Mexico and Hawaii winter assemblies are, at least, seasonally isolated. That is, whales leave northern feeding grounds and migrate to one winter assembly or the other, then after breeding success or seasonal residency, return to feeding areas. In 2016, the US National Marine Fisheries Service (NMFS) went significantly further, to designate the Mexico and Hawaii assemblies as Distinct Population Segments (DPS).^1^ The whales within each region were given different conservation status under the Endangered Species Act: Mexico ‘Threatened’ and Hawaii ‘Not at Risk’—inferring biologically separate entities [6,7].

This paradigm of separate humpback whale populations in the North Pacific emerged in the 1990s and 2000s. Regional and Pacific-wide photo-identification sampling indicated strong migratory preferences between specific feeding grounds and breeding areas, for example, Southeast Alaska and Hawaii or Pacific Northwest and Mexico [8–11]. Genetic studies found differences in haplotype frequency between the photo-ID sampled areas, including between the breeding grounds [12]. Both ID matching and genetic studies recognized ‘exceptions to these (migratory) patterns' and ‘potential high levels of plasticity in (whale) movements'. However, the generality became the basis of the DPS designations [6].

In fact, there are multiple lines of evidence of mixing between Mexico and Hawaii humpback whale populations. Individual whales interchange between Hawaii and Mexico from one winter to the next (e.g. [8–11,13–15]). Hawaii and Mexico populations share some and often all of the same phrases in their complex and changing song—something impossible without mixing at some point in annual cycles [16–19]. Whales from multiple feeding areas mix in one breeding area, (e.g. whales from Alaska, British Columbia and Russia in Hawaii); whales in a single feeding area may migrate to different breeding areas (e.g. whales from Russia found in Asia, Hawaii, Mexico) [9,13,14,20–22]. Pacific-wide song comparisons point to ocean basin scale interactions, not divisions [19]. A *same* winter match was reported, an individual whale being identified in the winter of 1986 in both Mexico (February) and Hawaii (April) [23]. Humpback whales have been detected acoustically within breeding seasons at latitudes midway *between* Mexico and Hawaii [24].

Here, we provide further direct evidence of mixing between Mexico and Hawaii humpback whale assemblies with the report of two more individually photo-identified whales that travelled between these regions in the *same* winter—that is, attended breeding assemblies in both the eastern and central North Pacific within a single breeding season.

## 2. Methods

### (a) Photo-identification

The photo-identification of individual whales by the unique and permanent skin pigment patterns on the underside of the flukes has, since the 1970s [25], become the basis for the majority of studies of humpback whale abundance, migrations, population definition and behaviour. Recently, very accurate recognition software and computerized matching programs have enabled large-scale comparison of tens of thousands of identifications and provide an increasingly detailed picture of humpback whale movement patterns in the North Pacific and worldwide [26]. This report is a result of Happywhale (www.happywhale.com) computerized matching efforts.

### (b) Effort

Overall, in its entire North Pacific matching project, Happywhale compared 26 607 humpback whale individuals from years 1977 to 2020 from 1851 contributors from locations spanning the ocean, including Mexico, Hawaii, Japan, the Philippines, California, Oregon, Washington, British Columbia, Alaska and Russia. Contributors of photo-identifications included long-term research projects, whale watch tour operators and the general public.

**Figure 1.**
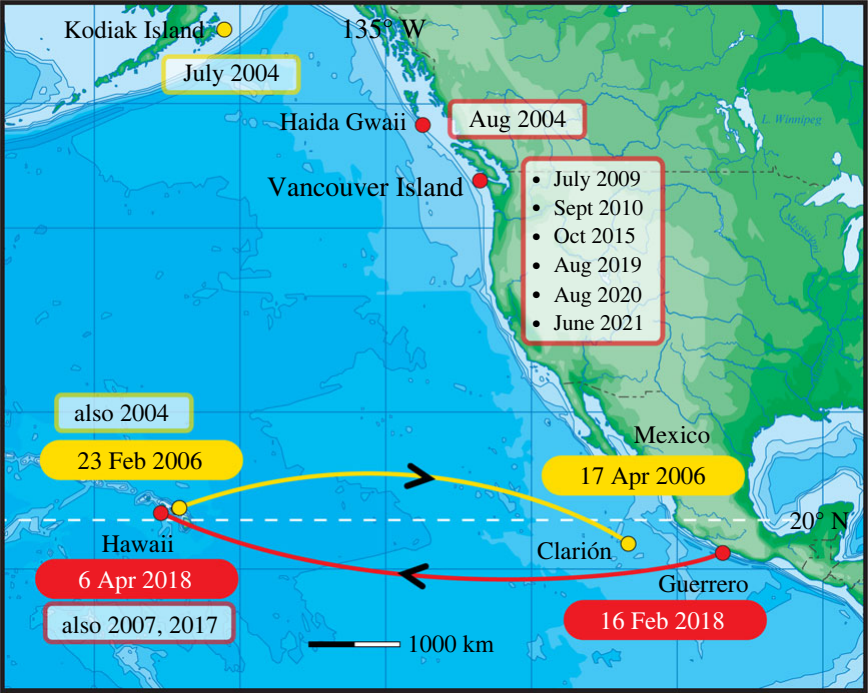
Records of two different individuals, which attended both Mexico and Hawaii breeding grounds in one winter, one in 2006 (yellow) and the other in 2018 (red). Additional sightings of these whales are outlined in the representative colour. The 2006 whale (yellow) was also identified off Kodiak Island, Alaska. The 2018 whale (red) was identified off British Columbia or northernmost Washington in seven summers.

This effort generated multiple sightings of the two individual whales pertinent to this report, listed in table 1 and summarized in figure 1. One individual was documented four times from 2004 to 2006 in three locations: Hawaii [2]; Mexico [1]; Kodiak Island, Alaska [1]. The second whale was identified 15 times between 2004 and 2021, in four locations: Hawaii [3]; Mexico [1]; northern British Columbia [1]; southern Bristish Columbia/northern Washington [11]. Of these 19 sightings (both cases combined), seven came from tour operators and 12 from research projects.

**Table 1.** Sightings histories of the individual whales found in both Mexico and Hawaii in the same winter season—Match 1 in 2006 and Match 2 in 2018. This information is also available for Match 1 at https://happywhale.com/individual/15116, and Match 2 at https://happywhale.com/individual/7270. HIHWNMS—Hawaiian Islands Humpback Whale National Marine Sanctuary, UABCS—Universidad Autónoma Baja California Sur, SWFSC—Southwest Fisheries Science Center, BC—British Columbia, WA —Washington State, USA, DFO—Fisheries and Oceans Canada, OSU—Oregon State University, WHET—Whale Habitat, Ecology and Telemetry Laboratory.

region	date	location		contributor information			
		detail	lat., long.	organization	ID label	contact (for ID collection)	photographer
2006 MATCH							
*winter*							
Hawaii, Maui	17 Mar 2004	West Maui	20.83, −156.70^a^	Whale Trust	HI04-0215	Meagan Jones, mjones@whaletrust.org	Charles Nicklin
Hawaii, Maui	23 Feb 2006	West Maui, Olowalu	20.75, −156.71	HIHWNMS	HI06-0311	Ed Lyman, ed.lyman@noaa.gov	Astrid Grupenhoff
Mexico, Revillagigedo	17 Apr 2006	Isla Clarión, South	18.34, −114.73	UABCS	UABCS-MN5-06R0366	Pamela Martínez, pamelapuma@gmail.com	Alberto Abad
*summer*							
Alaska, Kodiak Is.	30 Jul 2004	Kodiak East, Marmot Bay	57.72, −151.94	SWFSC	SWFSC-0327	Jay Barlow, jay.barlow@noaa.gov	Siri Hakala
2018 MATCH							
*winter*							
Hawaii, Hawaii/Big Island	29 Mar 2007	West Hawaii	19.94, −155.88	Eye of the Whale	EOTW-00-31	Beth Goodwin, bethogoodwin@yahoo.com	Beth Goodwin
Hawaii, Oahu	27 Apr 2017	West Oahu		Wild Side Specialty Tours			
Mexico, Guerrero	16 Feb 2018	Barra de Potosi	17.60, −101.55	Whales of Guerrero	WGRP-HB391	Katherina Audley, katherina@whalesinmexio.com	Whales of Guerrero/ Raul Ramírez
Hawaii, Maui	6 Apr 2018	West Maui, Olowalu	20.78, −156.62	HIHWNMS	HIHWNMS-2018-4-6- G08A04	Ed Lyman, ed.lyman@noaa.gov	Ed Lyman
*summer*							
BC, Haida Gwaii SE	15 Aug 2004	Moresby Is., Houston Stewart Ch.	52.47, −131.06	DFO	BCX0767	Thomas Doniol-Valcroze, Thomas.Doniol-Valcroze@dfo-mpo.gc.ca	Lisa Spaven
BC, Van. Is. SW	25 Jul 2009	Barkley Sd	48.88, −125.40^a^	Cascadia Research Collective	CRC15968	John Calambokidis, calambokidis@cascadiares.org	Wendy Szaniszlo
BC, Van. Is. SW	2 Sep 2010	Clayoquot Sd	49.06, −126.00^a^	Pacific Wildlife Foundation	CS413	Josie Byington, info@clayoquotwhales.ca	Peter Schulze
BC, Van. Is. SW	22 Oct 2015	Swiftsure Bank	48.60, −124.99	Ocean Ecoventures	BCX0767 ‘Flint’	Tasli Shaw, taslishaw@gmail.com	Tasli Shaw
BC, Van. Is. SW	26 Aug 2019	Swiftsure Bank	48.55, −124.77	Orca Spirit Adventures		Sarah Keenan, strait2sea@gmail.com	Sarah Keenan
BC, Van. Is. SW	7 Sep 2019	Swiftsure Bank	48.57, −124.81	Orca Spirit Adventures		Sarah Keenan, strait2sea@gmail.com	Sarah Keenan
BC, Van. Is. SW	10 Sep 2019	Swiftsure Bank	48.53, −124.87	Ocean Ecoventures	BCX0767	Gary Sutton, garysj27@gmail.com	Gary Sutton
WA, Olympic Pen. NE	19 Sep 2019	Swiftsure Bank	48.50, −124.86.	OSU Marine Mammal Institute WHET Lab	OSUWTG-MNWA-192	Craig Hayslip, craig.hayslip@oregonstate.edu Daniel Palacios, daniel.palacios@oregonstate.edu	Craig Hayslip
WA, Olympic Pen. NE	24 Sep 2019	Swiftsure Bank	48.50, −124.87	OSU Marine Mammal Institute WHET Lab	OSUWTG-MNWA-192	Craig Hayslip, craig.hayslip@oregonstate.edu Daniel Palacios, daniel.palacios@oregonstate.edu	Craig Hayslip
BC, Van. Is. SW	3 Aug 2020	Swiftsure Bank	48.52, −124.84	Orca Spirit Adventures		Sarah Keenan, strait2sea@gmail.com	Sarah Keenan
BC, Van. Is. SW	23 Jun 2021	off Port Renfrew	48.75, −124.95^a^	Orca Spirit Adventures		Matt Burnaby, strait2sea@gmail.com	Matt Burnaby

^a^Latitude/longitude approximate.

## 3. Results

**Figure 2.**
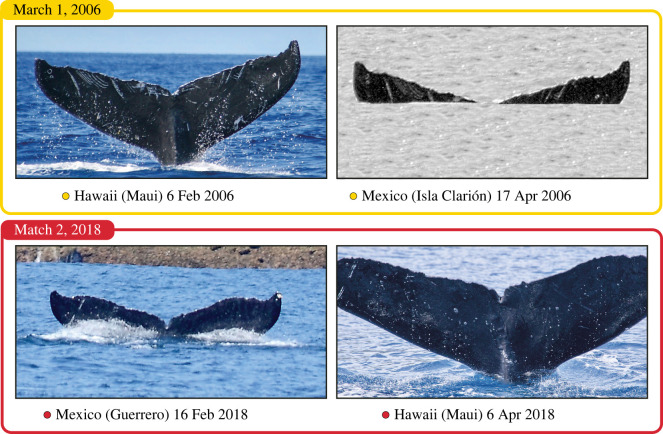
Photo-identifications of the individual whales that travelled between Mexico and Hawaii in one winter.

The sightings histories of the two whales that travelled between Mexico and Hawaii in one winter are listed in table 1 and illustrated in figure 1. Figure 2 shows the photographic identification matches.

### (a) Hawaii–Mexico Match 1 (2006): Hawaii 23 February 2006 to Mexico 17 April 2006, male

This individual was identified on 23 February 2006 off Olowalu, on the west side of the island of Maui. It was in a surface-active group of 5–7 animals. These groups typically consist of multiple males and one female. This matched whale was identified as the principal escort (PE) to the female at the time of the sighting, indicating the animal was a male.

Then, on 17 April 2006, 53 days later and 4545 km distant, it was identified off Isla Clarión in the Revillagigedo Archipelago, Mexico. At that time, this individual was one of a trio of whales.

#### (i) Other sightings

There are two additional sightings of this whale. One was in Hawaii, off West Maui, on 17 March 2004, two winters before the Hawaii–Mexico travel. At the time it was singing, a male behaviour. This whale was also identified in a summer feeding ground off Kodiak Island, Alaska on 30 July 2004.

### (b) Mexico–Hawaii Match 2 (2018): Mexico 16 February 2018 to Hawaii 6 April 2018, male

This individual was identified on 16 February 2018 south of Zihuatanejo, Guerrero, Mexico. It was alone at the time, travelling rapidly with several breaches and a tail throw noted. There were two other whales in the general vicinity, and it is possible the encounter came after an interaction with one or both. This was the only sighting of this individual off Guerrero, and in Mexico, in the 2018 season.

Then, on 6 April 2018, 49 days later and 5944 km distant, this whale was identified in the Auau Channel off West Maui near Olowalu. It was one of seven whales pursuing a female in a surface-active group and very likely a male (more than one female in one of these groups does occur but is rare). It was observed for 40 min (14.30–15.10 HST) with no indication that it was the PE. It was one of the secondary escorts or challengers in the group. This was the only identification of this whale in Hawaii that season.

#### (i) Other sightings

Beyond the match year (2018), this whale has a relatively extensive sightings history, with 13 additional identifications in 9 of 17 years from 2004 to 2021. Two of these additional sightings were in Hawaii, the remainder in British Columbia or northern Washington.

In Hawaii, this whale was also identified on 29 March 2007 off the Kohala Coast of the Big Island of Hawaii, and on 27 April 2017 on the west side of the island of Oahu.

In British Columbia, the first sighting, and earliest record, of this whale was near the southern end of Haida Gwaii (a.k.a. Queen Charlotte Islands) on 15 August 2004. The next two identifications were on the central west coast of Vancouver Island, British Columbia, on 25 July 2009 in Barkley Sound and on 2 September 2010 in the adjacent Clayoquot Sound. The next eight sightings were between 2015 and 2021 off southwest Vancouver Island and northwest Olympic Peninsula on or near the Swiftsure Bank (entrance to Straits of Juan De Fuca): in 2015 on 22 October; in 2019 on 26 August and 7, 10, 19, 24 September; in 2020 on 3 August; and in 2021 on 23 June (table 1).

The British Columbia locations are all on feeding grounds, and other than the 2004 Haida Gwaii record, all were within 200 km of each other. The Haida Gwaii sighting was approximately 800 km further west.

This whale's age, assuming it was at least a yearling in 2004 (that is, it was not identified then in a mother–calf pair), would be, at the time of the Mexico–Hawaii match, a minimum of 15 years old, and it was likely sexually mature [27].

#### (c) Travel times

It is not possible to determine actual travel times since we cannot know date of departure from one assembly area or date of arrival in the other. Nor can we know if travel was direct and steady or if whales lingered at some point between the departure and destination. However, rough calculations can be made which suggest a range of travel times as shown in table 2. It is unlikely that whales were photo-identified the day they departed and the day they arrived so the ID to ID are likely overestimates of travel time (in days). Migration travel speeds in the literature range from about 4 km h^^−1^^ to the fastest speed found of 7 km h^^−1^^ [28–33]. Calculations using these values led to broad estimates of travel time between Hawaii and Isla Clarión in the Revillagigedo Archipelago in 2006 (Match 1) of 27–47 days, and mainland Mexico and Hawaii in 2018 (Match 2) of 35–62 days. The Mexico and Hawaii identifications of the 2018 (Match 2) whale were 49 days apart, and the calculated travel time at 4 km h^^−1^^ is 62 days, so it apparently travelled at a higher speed than that.

**Table 2.** Estimates of Hawaii–Mexico travel times. Speeds of travel used in the calculations came from: (1) satellite tags in the North Pacific 4.5-6.2 km h^^−1^^ [28] and an average of 4 km h^^−1^^ [29], in the South Pacific 3.53 ±2.22 km h^^−1^^ [30], and in the South Atlantic the fastest speed recorded at 7 km h^^−1^^ [31]; (2) a North Pacific migration photo-identification match [32] indicating 4.79 km h^−1^ the minimum speed; and (3) the measurement of migratory speed off eastern Australia [33], a range depending on behaviour but with a mean of 4 km h^−1^.

whale	Mex. date	HI date	direction	distance (km)	travel time (days)		
					ID to ID (speed)^a^	@ 4 km h^−1^	@ 7 km h^−1^
1986^b^	5 Feb 86	27 Mar 86	E to W	4700 Clarión–Kauai	51 (3.8 km h^−1^)	49	30
Match 1	17 Apr 06	23 Feb 06	W to E	4545 Maui–Clarión	53 (3.6 km h^−1^)	47	27
Match 2	16 Feb 18	7 Apr 18	E to W	5944 Guerrero–Maui	49 (5 km h^−1^)	62	35

^a^Travel speed if whales were photo-identified the day they departed and the day they arrived.

^b^From Forestell & Urbán [23].

## 4. Discussion

Individual humpback whales (at least males) may travel between the distant (4500–6000 km apart) Mexico and Hawaii breeding assemblies in the same winter season. These travel records are consistent with the recent, mid-ocean detection of humpback whale songs in winter between the locations [24]. In fact, this mid-ocean detection occurred in 2018, the year of the Guerrero–Hawaii travel. The delineation of these traditional winter grounds has become less clear in that not only may humpback whales attend both regions during a winter breeding season, they may also be present over a broad reach of the tropical North Pacific, between Hawaii and Mexico at that time.

The direct, within-season travel records between Mexico and Hawaii bolster^2^ an earlier, similar report [20]—and provide two new insights. The first is that, not only do whales travel east to west, from Mexico to Hawaii, but they also travel west to east, Hawaii to Mexico, in one season. The second insight is that not only do whales travel between Hawaii and the westernmost Mexico breeding habitat at Isla Clarión in the Revillagigedo Archipelago, but also between Hawaii and southern mainland Mexico, some 1000 km further distance.

We are not aware of other examples of same-season connectivity between humpback whale breeding grounds isolated by large longitudinal distances. However, several instances of same-season movement between breeding assembles have been reported in the southwestern Pacific [34]. Direct comparison of this behaviour between the northeast and southwest Pacific is complicated by the relatively contiguous island chains (that is, breeding habitat) in the south versus the 4500–6000 km of deep ocean between Mexico and Hawaii in the north. Nonetheless, the South Pacific observations do indicate that humpback whales may attend more than one geographically defined breeding ground in one season.

Beyond the Mexico–Hawaii connections are the multiple summer sightings of the 2018 Match 2 whale (in 7 of the 18 years from 2004 to 2021) off British Columbia, with sightings in six of those years in the same locale off the southwest coast of Vancouver Island. The location of this whale in the other 11 years is not known. Notably, this whale was not identified in the summer that followed the Hawaii 2017 sighting, or in the summer following the 2018 Mexico–Hawaii match, but was again found in this Vancouver Island location in summer 2019, 2020 and 2021. Humpback whales identified in this one summer feeding ground off southwest Vancouver Island (along less than 200 km of coast) have been found in all four DPS designated breeding grounds: Japan, Hawaii and Mexico, and Central America [10,11,14,21,22] and in the case of this report, two of these grounds in one season. These observations support the hypothesis of a level of fidelity to specific feeding grounds [12,35], but, at the same time, suggest there is potential for widespread mixing during the winter breeding season.

The single record of the 2006 Match 1 whale in a feeding ground off Kodiak Island, Alaska indicates that it is not only whales from a specific feeding area off British Columbia that may attend Hawaii and Mexico breeding grounds in one winter.

The same-season Mexico–Hawaii travels equate with observations of song sharing [16–19], and interchange of individual whales year to year (e.g. [9–11,13]). Together these studies indicate decades-long interaction between whales that use these two winter regions. Consistent with this view are the observations of whales from one feeding area migrating to both (and more) breeding locations, where they mix with whales from other feeding grounds [13,14,21,22]. This collective evidence would lead logically to a hypothesis of one panmictic, or several highly overlapping, humpback whale populations in the northeast Pacific—something that was initially proposed in the late 1970s [17].

While the application of the DPS designations may serve an important role for the US Endangered Species Act, real questions arise as to whether these designations, in current form, are a useful reflection of the biology of North Pacific humpback whales. In the formation of these DPS, a number of factors were not given weight, or even considered: multiple breeding ground destinations from one feeding ground; mixing of whales from multiple feeding grounds in one breeding ground; year to year interchange between breeding grounds; and song sharing. Further, the data most influential in DPS designation, the genetic comparisons [12], may be open to question. They were based on the ‘*a priori*’ determination of the groups to be compared, known to result in the recognition of artificial genetic differentiation between groups when none may actually exist^3^ [36–40].

Evidence of mixing between the whales that compose the Mexico and Hawaii populations is indisputable; the question now is one of significance. Is the mixing a rare occurrence with negligible biological impact or management consequence, or is it a reflection of a biologically meaningful integration of humpback whales throughout the northeast Pacific—if not the entire ocean basin?

## Endnotes

1 The DPS is a US Endangered Species Act legal designation for the purpose of listing, delisting and reclassifying vertebrates. It is the smallest division of a species protected under the Act. Three elements are considered sequentially in determining the status of a potentially distinct population segment: '(1) the discreteness of the population relative to the rest of the species; (2) the significance of the population segment to the species; and (3) the population segment's conservation status in relation to the Act's standards for listing (i.e. is the population segment endangered or threatened when treated as if it were a species?)' [5, p. 4725].

2 This was the only report of same-winter travel between Mexico and Hawaii, and the NMFS photo-identification matching program (in the 1980s) that found the match could not verify the Hawaii date to its satisfaction. However, this observation was published (17 years later)[23], and the findings reported in the current paper make it much less of an outlier.

3 This is a well-known problem in population genetics (e.g. [36,37]), and is the main reason why emphasis has shifted away from assessing population structure using methods that require *a priori* groupings towards those that do not (e.g. [38,39]. This reliance on *a priori* groupings is especially a problem when a population shows a pattern of ‘isolation by distance': where there is one population but also a positive relationship between genetic and geographic distance [40], which, based on the figures in [12], may be the case.

## Data accessibility

The data on which this paper is based is accessible at https://happywhale.com/individual/15116, and https://happywhale.com/individual/7270. The key data is all included in the main body of the paper. These are the photo-ID photographs in figure 2. Also, figure 1 and table 1 list other (peripheral) sightings (and photo-identifications) of the same whales. The actual identification photographs of the peripheral sightings, along with date and location information, are available at the links above. The ID photographs are contributed to www.happywhale.com by research groups and whale watch companies. The ID photograph comparisons, leading to the sightings history of individual whales, are made with a computer matching program then checked visually. As shown in the examples in the paper the matches are clear, that is, there is enough information on the identification photographs to be certain they are the same whales. This is a standard technique used in whale research programs worldwide. A detailed description of the computerized matching process in given in [26].
